# Power Transformers OLTC Condition Monitoring Based on Feature Extraction from Vibro-Acoustic Signals: Main Peaks and Euclidean Distance

**DOI:** 10.3390/s23167020

**Published:** 2023-08-08

**Authors:** Fataneh Dabaghi-Zarandi, Vahid Behjat, Michel Gauvin, Patrick Picher, Hassan Ezzaidi, Issouf Fofana

**Affiliations:** 1 Research Chair on the Aging of Power Network Infrastructure (ViAHT), Department of Applied Sciences (DSA), University of Quebec at Chicoutimi (UQAC), Saguenay, QC G7H 2B1, Canadavbehjat@etu.uqac.ca (V.B.); hezzaidi@uqac.ca (H.E.); 2 Hydro-Québec’s Research Institute (IREQ), Varennes, QC J3X 1S1, Canada

**Keywords:** power transformer, OLTC, vibro-acoustic signals, main peaks

## Abstract

The detection of On-Load Tap-Changer (OLTC) faults at an early stage plays a significant role in the maintenance of power transformers, which is the most strategic component of the power network substations. Among the OLTC fault detection methods, vibro-acoustic signal analysis is known as a performant approach with the ability to detect many faults of different types. Extracting the characteristic features from the measured vibro-acoustic signal envelopes is a promising approach to precisely diagnose OLTC faults. The present research work is focused on developing a methodology to detect, locate, and track changes in on-line monitored vibro-acoustic signal envelopes based on the main peaks extraction and Euclidean distance analysis. OLTC monitoring systems have been installed on power transformers in services which allowed the recording of a rich dataset of vibro-acoustic signal envelopes in real time. The proposed approach was applied on six different datasets and a detailed analysis is reported. The results demonstrate the capability of the proposed approach in recognizing, following, and localizing the faults that cause changes in the vibro-acoustic signal envelopes over time.

## 1. Introduction

Power transformers are key components in power transmission and distribution networks and, therefore, it is very important to ensure their safe and reliable operating condition [1,2,3]. An On-Load Tap-Changer (OLTC) is one of the most important components of the power transformer and it is used to regulate the transformer voltage by changing the number of turns in one winding of the transformer (turns ratio). It is reported that around 30% of transformer faults are due to OLTC failures, hence the requirement for a timely condition assessment of this component [4,5,6].

Most OLTCs are based on the mechanical switching principle for the selection of the tap position without any interruption in the current. If OLTC uses a single contact switch during a tap-change operation, the current would definitely be cut-off until the switch was connected to the final tap position. To solve the current cut-off problem, the “make before break” contact concept is used. In this regard, a final tap position is bridged before breaking contact from the primary tap position. So, the OLTC switch connects the two taps at the same time and then a circulating current is created. To limit the circulating current, transition impedance is used in the form of a resistor or reactor [7,8,9].

There are several techniques adopted for the condition monitoring of an OLTC, such as dynamic resistance measurement (DRM), dissolved gas-in-oil analysis (DGA), vibro-acoustic signals analysis, motor-drive current analysis, etc. [10]. In this research work, vibro-acoustic signals analysis is used to evaluate the OLTC condition. This method is used as an on-line and real-time method, in which the faults are detected by analyzing vibro-acoustic signals generated from OLTC components.

Several approaches have been proposed to detect OLTC faults using vibro-acoustic signal envelopes [11,12,13,14,15,16]. The authors of references [15,16] presented a method in which a base waveform is selected by Spearman’s Rank correlation coefficient, and the condition of each OLTC tap position is evaluated by comparing its waveform with the base waveform. In reference [17], all vibro-acoustic signals of the OLTC are split into two parts. Each part is decomposed into 16 sub-components using wavelet packet transform, and the energy entropy of each sub-component is computed to achieve the condition assessment. In several works [11,12,13,14], the OLTC condition is evaluated based on the main peak extraction from vibro-acoustic signal envelopes. For example, in reference [12], the bursts are extracted based on the vertical maximum lines of the continuous wavelet transform (CWT).

It should be noted that each generated vibro-acoustic signal envelope from OLTC consists of several main peaks. In this regard, information about the number of main peaks, their amplitude, and the time delay between individual peaks will help to detect OLTC faults in the early stage [11]. Over time, changes in the amplitude of main peaks and the time lag between the peaks indicate faults in the components that include springs, switching contacts, and gears [18].

The authors of reference [19] describe how the OLTC faults, including contact wear and weak spring, generate vibro-acoustic signal envelopes with higher amplitudes and increased transition time between peaks. The OLTC faults, including tap loosening, tap loss and tap abrasion, cause some changes in the amplitude of the bursts [20].

It is generally believed that an analysis of the main peaks of the vibro-acoustic signal envelopes is a promising approach to evaluate the OLTC condition. However, extracting the main peaks from the signal envelopes is a challenging task. The first challenge is the discrimination of the main peaks’ locations, which may be very close to each other. On the other hand, and as another major challenge in main peaks extraction, there may be a variable time shift between the main peaks that occur due to reasons such as gradual contact wear and this displacement makes main peak extraction a challenging task.

To simplify the signal analysis, averaging and time realignment techniques are used to reduce the natural variations between vibro-acoustic signal envelopes of a tap-changer in good conditions [21,22]. Trending and instantaneous algorithms are used to detect long-term changes and the sudden variation in vibro-acoustic signal envelopes [19]. An index can be used to detect changes over time and provide a metric to compare data related to several sister OLTCs [23].

The current work aims to develop a methodology for the condition monitoring of OLTC based on vibro-acoustic signal envelopes. The proposed method focuses on detecting, localizing, and tracking changes in vibro-acoustic signal envelopes utilizing main peaks extraction and Euclidean distance (ED). However, there are two sub-objectives to attain the major objective of this research. The first is to extract the main peaks from vibro-acoustic signal envelopes. This sub-objective is covered by utilizing the concept of dynamic and static windows which address the amplitude of the main peaks and the zones containing the main peak locations. The second sub-objective is to compute ED between each envelope and a reference envelope. This metric is calculated using the amplitude of the main peaks of each envelope in comparison to the reference envelope. It should be noted that this distance is not computed for the whole signal envelope. The calculation is carried out for all the main peaks existing in the envelopes. In addition, the amount of ED is also calculated for each zone located in each envelope, in comparison with the reference envelope. This metric distance is used to track changes in vibro-acoustic signal envelope patterns over time.

## 2. Data Collecting and Pre-Processing

### 2.1. Data Collecting

**Figure 1:**
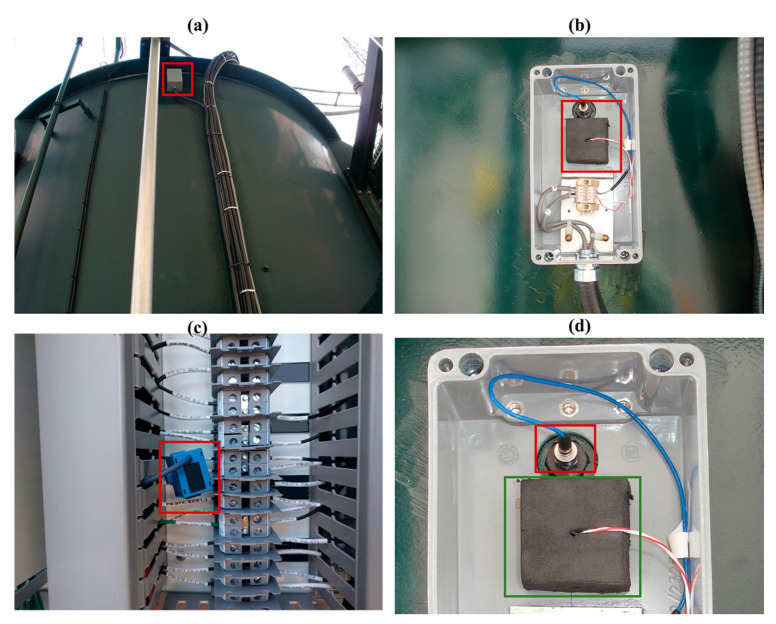
Installation of the accelerometer, temperature, and the current clamp sensors: (**a**) the box where the accelerometer and temperature measurements are installed is highlighted with a red rectangle; (**b**) a zoomed view of the accelerometer and temperature measurements in the box; (**c**) the current clamp sensor; (**d**) the accelerometer and temperature measurement sensors (red and green rectangles, respectively).

**Figure 2:**
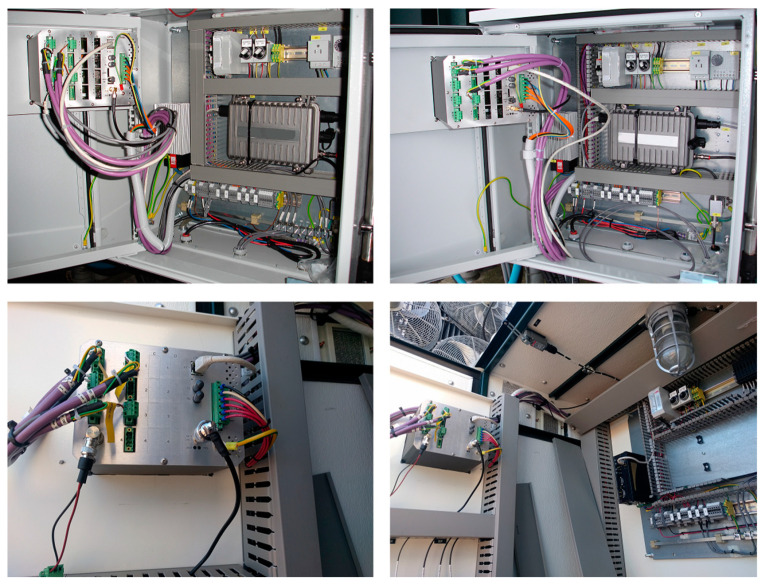
The TMU and other related equipment.

There are more than 2000 transformers with an average age of nearly 30 years in the Hydro-Québec’s network, and approximately half of them are equipped with OLTC. To optimize the maintenance strategies of the transformers, the Hydro-Québec’s Research Institute (IREQ) started a research project to monitor power transformers in OLTC. In this regard, the IREQ’s technology was industrialized and OLTC monitoring systems were first installed on nine single-phase autotransformers rated 370 MVA, in a service on the Hydro-Québec’s electric network [23]. The details about the functional architecture of this monitoring system are explained in reference [21]. This monitoring system measures vibro-acoustic signals and motor current using an accelerometer installed on the surface of the transformer tank near the diverter switch (for OLTC placed in the main transformer tank), and a current clamp around the wire powering the OLTC’s motor, respectively. In addition, to characterize the effect of the temperature on the vibro-acoustic signal, the temperature is also measured and recorded on the surface of the tank near the accelerometer. In Figure 1a, the accelerometer and temperature measurement sensors are highlighted with a red rectangle. Figure 1b shows a zoomed view of the accelerometer and temperature measurement sensors in the box. In Figure 1c, the current clamp sensor is highlighted using a red rectangle. In Figure 1d, the accelerometer and temperature measurement sensors are highlighted using red and green rectangles, respectively. The back of the transformer monitoring unit (TMU), which records the measurements, is shown in Figure 2. This TMU computes, saves, and transmits, via a communication system, the envelopes of the motor current and vibro-acoustic signal, and other parameters such as the tap position and the temperature. The TMU’s output can also be correlated with other data such as the transformer load and the transformer top-oil temperature.

This research studies the vibro-acoustic signal envelopes, specifically the switching operation portion of the signal of three OLTCs corresponding to three sister units which have been monitored continuously from 2016 to 2021. The transformers under study are referred to as T3A, T3B, and T3C hereafter in this paper. It should be noted that the in-tank brand and model of these OLTCs is ABB UC. According to the mechanical design of their main switching contacts (odd and even position) [23], it is possible to divide each dataset into these two categories, odd and even positions. This brings us to analyze six datasets that are named T3A-odd, T3A-even, T3B-odd, T3B-even, T3C-odd, and T3C-even.

### 2.2. Pre-Processing

The raw measured vibro-acoustic signals are complex, not aligned, and, therefore, not suitable for extracting useful information and enabling trending. In this work, two pre-processing techniques are applied on vibro-acoustic signals. The first pre-processing is signal envelope extraction. The original vibro-acoustic signals are complex and non-linear and should be simplified to provide better analysis possibilities. One of the most common approaches that is used to simplify signals is signal envelope extraction. This approach generates a smooth signal based on the original signal. There are several filters and methods, such as Hilbert transform, low pass filter (LPF), moving average (MA), wavelet transform, Savitzky–Golay filter, and Hilbert–Huang Transform (HHT), that are used to extract a signal envelope [15,16,20,24,25]. In this research, Hilbert transform and LPF are used to extract the signal envelope. It should be noted that this pre-processing computing is carried out in the TMU and just the signal envelope is saved. The second pre-processing is a signal envelope alignment. Temperature and other parameters affect the vibro-acoustic signal envelopes and create a time lag between them. Therefore, vibro-acoustic signal envelopes should be aligned to simplify comparison. One of the techniques is a first-order time-realignment technique which is used in this paper [21,22]. In addition, a moving average of ten consecutive envelopes is used to reduce the natural variations between vibro-acoustic signal envelopes. Moreover, a reference is established for each OLTC dataset individually and is achieved by computing the average of twenty envelopes starting from the commissioning of the TMU. For best results, this reference should be taken when the OLTC is in good condition, when it is new or after a maintenance or an inspection confirming its good state. Comparing measured envelopes over time with a reference helps to detect changes over time.

**Figure 3:**
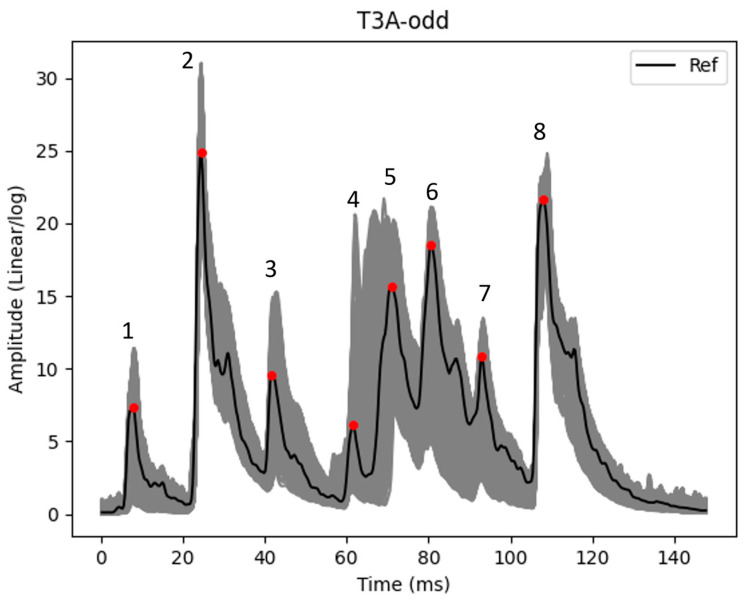
The reference and all averaged and aligned envelopes (in grey) related to dataset T3A-odd. The eight main peaks of the reference are highlighted with red circles.

Figure 3 shows the reference related to dataset T3A-odd and all the envelopes of the dataset (shown in grey). It should be noted that the envelopes are processed using the alignment technique. In addition, combined logarithmic and linear scales are also applied on vibro-acoustic signal envelopes.

## 3. Extracting Main Peaks

**Figure 4:**
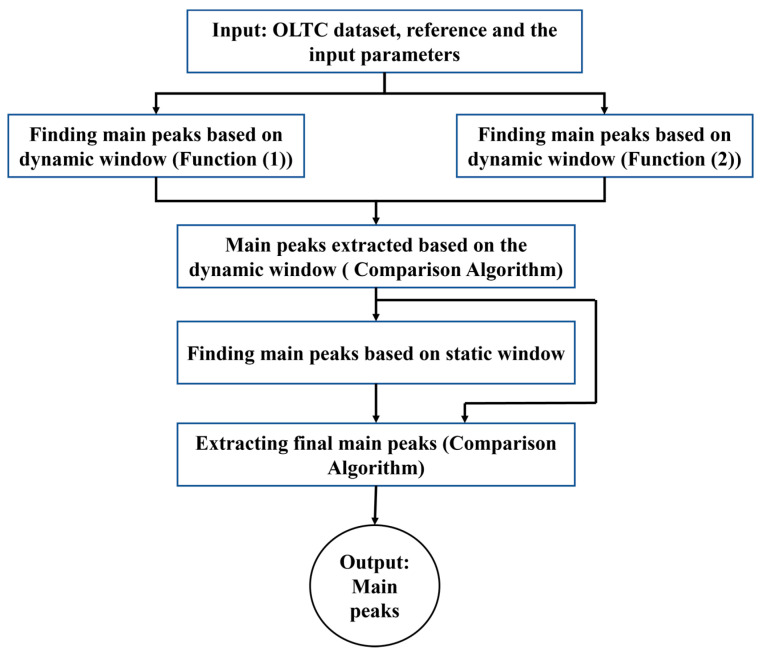
The steps to identify the main peaks of vibro-acoustic signal envelopes.

As mentioned, there is a reference for each dataset. In the first step, the main peaks of the reference are extracted. It should be noted that there are many peaks in the reference, and the peaks with the maximum local amplitude over a zone are highlighted as the main peaks. In Figure 3, the eight main peaks of the reference are highlighted using red circles. According to the time index of each reference main peak, the main peaks in all envelopes (with respect to the time) are extracted based on some window functions and comparison algorithms. Figure 4 shows the steps to identify the main peaks. These steps are carried out for each major peak identified in the reference (see Figure 3), individually. In this regard, dynamic windows are applied two times using different functions. It should be noted that functions (Function (1) and Function (2)) are applied parallel in order to find main peaks based on dynamic window. A comparison algorithm is then applied to select the best position of the peaks obtained from the dynamic window functions. Then, the main peaks are extracted based on a static window function in order to extract better results. The size of the static window is adapted based on the main peak location extracted from the dynamic window and comparison algorithm. In the flowchart depicted in Figure 4, it can be clearly observed that the final peak detection block branches from both static and dynamic blocks. In fact, a comparison algorithm is applied to the results from the static and dynamic windows to extract the final localization of the main peaks. In the following, the dynamic and static window functions and comparison algorithm are explained in detail.

### 3.1. Dynamic Window

The dynamic windows slide over the zones of the main peaks with a size less than 20 samples. This small sample size minimizes the chance of wrong main peak positioning, especially in areas where there are multiple main peaks located close to each other (see, for instance, main peaks 4, 5, 6, and 7 in Figure 3). The windows’ moving ability enables the capability to track the time displacement of the main peaks (see, for instance, the main peak 5 in Figure 3). These windows are used individually for each main peak identified in the reference.

The middle of the dynamic windows is located at the index of the reference main peaks. Then, all existing peaks within the windows are found and one of these peaks is considered as the main peak. It should not go without saying that this procedure is faced with two challenges, including the absence of peaks in the window area and the identification of one peak as the main peak among several peaks. In the following, each of these issues and the provided solutions are explained in detail.

#### 3.1.1. Absence of Peaks in the Dynamic Window Area

**Figure 5:**
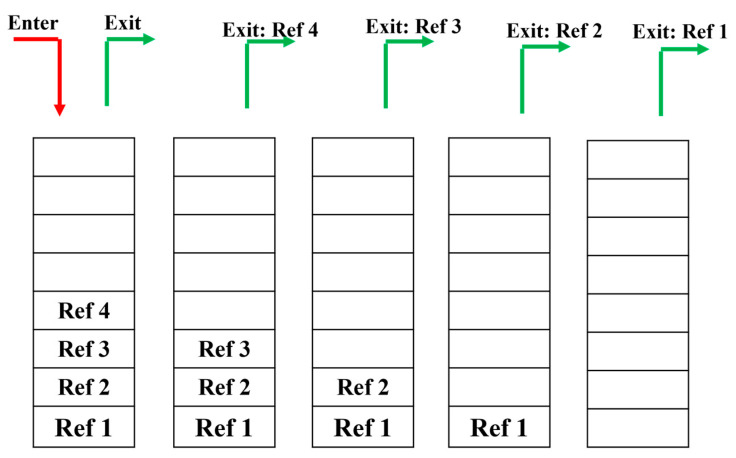
LIFO stack.

As mentioned, a dynamic window has a sample size smaller than 20 samples and it is normal that there are no peaks within the window. Increasing the size of the dynamic window would be more likely to lead to identifying the wrong location for the main peak. Therefore, we use a last in, first out (LIFO) stack [26] to store the index of found main peaks. When the main peak in a window is found, the position of this peak is stored in this stack (push operation). This step is carried out for the next envelopes with respect to time. In the procedure of finding peaks in envelopes, if there are no peaks within a window, the index of the corresponding reference peak is changed with the index stored in the stack. As such, the last index position entered in the LIFO stack is extracted and used as the reference index (pop operation). If there is still no peak in the window, the reference index of the last location in the LIFO stack is used. This process continues until one peak is found or the stack is empty. Figure 5 represents this procedure. As depicted in this figure, the main peak was identified in four envelopes and their peak indexes were saved (push operation) in the LIFO stack. In this stack, Ref 4, Ref 3, Ref 2, and Ref 1 are, respectively, the last, third, second, and first index of the main peak that were found. Now, the main peak from the fifth envelope should be found. In this envelope, there is no peak in the window. Therefore, the latest index stored in the stack (Ref 4) (pop operation) replaces the reference index. If the peak is not found in the window again, the reference is changed with Ref 3. This step continues until a peak is found, or the stack is empty. If the stack is empty and no peak is found, it is considered that there is no peak in this area of the fifth envelope.

#### 3.1.2. Selecting the Best Position for the Main Peak among Several Peaks

Generally, if there is more than one peak within the window, the peak that has a higher amplitude is considered the main peak. It should be noted that this assumption is not always true due to noise and natural variations between acoustic signatures of a tap-changer in good condition. As such, if there is more than one peak within the window, the two peaks (also referred to as separated peaks) that have the first and second highest amplitudes are identified and, later, one of them is selected as the main peak. Two functions, EF1 and EF2, are used to select the best position of the main peak between the two identified peaks. These functions represented in Equations (1) and (2) are computed between each identified peak and the reference peak.

(1) EF1= ED+MSE

(2) EF2=1/ED + AP

(3) $$\;\mathrm{ED}\;\left(X_{1},X_{2}\;\right)=\sqrt{{\left(x_{1}-{\;x}_{2}\right)}^{2}+{\left(y_{1}-{\;y}_{2}\right)}^{2}\;}$$

(4) $$\;\mathrm{MSE}=\frac{1}{n}\;\overset{n}{\underset{i=1}{\sum }}{\left(\mathrm{env}1\left(i\right)-\mathrm{env}2\left(i\right)\right)}^{2}$$

where ED is the Euclidean distance between the maximum value of the reference and identified peaks and MSE represents the mean square error between the peaks [11]. AP in (2) represents the amplitude value of the peak. It should be noted that MSE is computed for the area (10 samples) around the maximum value of the identified and reference peaks. n is the number of samples in env1 and env2, which is equal to 10 for the purpose of this study. These two evaluation functions (EF1 and EF2) are calculated for each of the two identified peaks, individually.

Following the EF1 function, a peak that has a lower EF1 value (smaller ED and MSE from the reference) is selected as the main peak. Based on the EF2 function, a peak that has a higher EF2 value is selected as the main peak.

If there is a difference between the results obtained using the EF1 and EF2 functions, the comparison algorithm explained in Section 3.3 is used to select the best index for the main peak.

### 3.2. Static Window

Because the dynamic window has a small sample size (less than 20 samples), it is likely that the main peak is located out of this window and that the wrong place is selected for the main peak. In addition, the risk of selecting the wrong position based on dynamic windows, is also possible. For instance, the peaks that are close to the main peak are likely to be mistaken as a main peak. A static window is introduced to solve these issues. For each main peak, a static window with a fixed time range (zone) is defined and the main peak is constrained to be located in the static window. For example, in Figure 3, and for the main peak 1, the size and location of the corresponding static window should consist of a time range around main peak 1 that the position of the peak in each envelope is placed in this range. As such, this window has a specific size for each main peak and its size is selected automatically for each main peak. The static window size (SWS) is computed for each main peak individually after applying the dynamic window functions for each main peak. SWS for each main peak can be computed according to (5).

(5) SWS= HTP−LTP+2

where HTP and LTP are the highest and lowest time positions of main peaks that are found in all envelopes after applying dynamic windows and the comparison algorithm, respectively. The start and end point of the static window is located at (LTP − 1) and (HTP + 1), respectively, and a peak that has the highest value in the static window is selected as the main peak. It should be noted that this window can be used if there is no overlapping with the neighbor’s main peak. In the considered datasets of this study, no overlapping is observed between the main peaks that are adjacent to each other.

### 3.3. Comparison Algorithm

**Figure 6:**
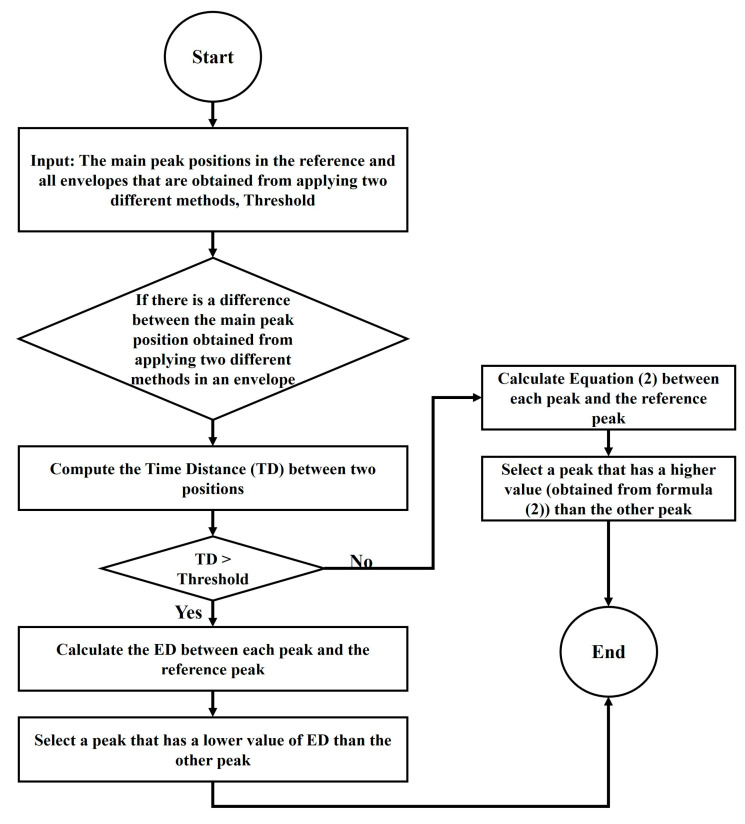
The steps of comparison algorithm.

This algorithm is used to select the best position for the main peak if there is a difference between the peak positions identified using the dynamic and static windows. To summarize, the main peaks are first identified using dynamic windows and functions EF1 and EF2. If there is a difference between the results obtained from these two functions, the comparison algorithm is used to select the best position. Subsequently, the main peaks are extracted using the static window. The comparison algorithm is applied a second time if there is a difference between the results obtained from dynamic and static windows (Figure 4). The steps of this algorithm are explained in Figure 6.

## 4. Computing ED

ED is a metric distance that is used to measure the amount of similarity between two points or two shapes. This metric is sensitive to shifting and, therefore, it is a good metric to track changes in vibro-acoustic signal envelopes. In this study, ED values are computed using data from each envelope in comparison with the reference signal envelope. It should be noted that ED is not computed for the whole signal. Two approaches are used. The first set of EDs is calculated using Equation (3) and it uses the main peaks localization (sample number and amplitude). The second set of EDs uses amplitude vectors in the area (zone) of the main peaks. This metric is calculated using Equation (6). The same number of EDs is calculated for both approaches.

(6) $$\mathrm{ED}2\;\left(T,\;S\right)=\sqrt{\overset{n}{\underset{i=1}{\sum }}{\left(T_{i}-{\;S}_{i}\right)}^{2}}$$

where T and S are vectors of length n (n is the number of samples in the zone). In the developed fault detection approach, the average value of each computed ED (for the amplitude of the main peak/zones) is calculated per year. Finally, the linear regression technique and R^2^ are used to identify the parts of envelopes where significant changes have taken place over time.

## 5. Results

In this section, the performance of the proposed algorithms is analyzed based on the implementation in Python 3.9.5 (PyCharm was used as the IDE). The peaks in the signal envelopes are extracted using the “find_peaks” function of the SciPy signal processing package.

**Figure 7:**
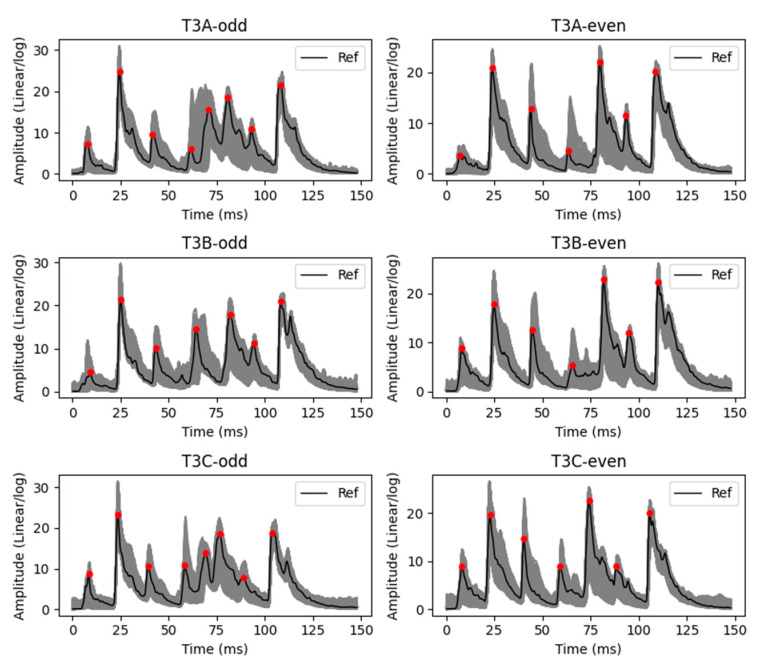
All envelopes in the datasets T3A-odd, T3B-odd, T3C-odd, T3A-even, T3B-even, and T3C-even are shown in grey. In addition, the references and their main peaks are displayed with solid black line and red circles, respectively.

In addition, six individual datasets are used to analyze the proposed methodology. It should be noted that, in the pre-processing stage of the signal envelope alignment, the envelopes are aligned and limited to 500 samples in each dataset. Figure 7 shows all envelopes in each of these datasets that have been measured from 1 September 2016 to 1 September 2021, individually in grey. In addition, in this figure, the reference of each dataset and the amplitude of main peaks of reference are displayed with a solid black line and red circles, respectively.

As mentioned, the first objective is to extract the main peaks from each envelope in each dataset. After that, in each dataset, the ED between the amplitude of each main peak in the reference and each envelope is calculated. In addition, it is possible to subdivide each dataset into the main zones based on the location of main peaks. The sizing and localization of each zone is achieved using the static window strategy presented in Section 3.2. After that, the ED is calculated between each zone in the reference and envelopes. In the following, the results related to the main peaks, zones, and ED are shown. It should be noted that the results of one dataset are described in this work to avoid repetition. Among the six datasets, the T3A-odd dataset was selected in this work because significant changes have been seen.

### 5.1. Extracting Main Peaks from Envelopes

**Figure 8:**
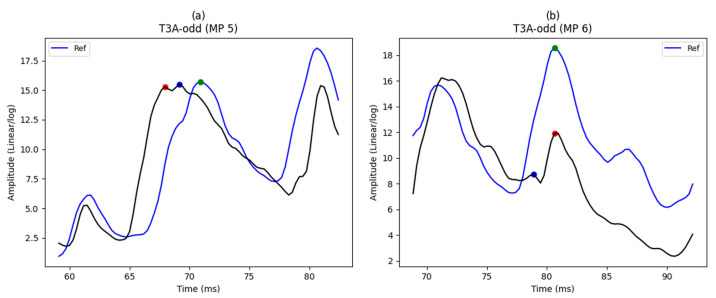
The results of two instance envelopes in the T3A-odd dataset: (**a**) main peak locations extracted using dynamic windows; (**b**) main peak locations extracted using dynamic windows and static window. A solid blue line represents the reference and its main peak and green dots, while the main peak positions extracted using dynamic windows are represented by red and blue dots on the envelope (black curve).

In this section, the main peaks are extracted (see Figure 4). To visualize the extraction of main peaks, the results of one envelope for main peak 5 and another for main peak 6 are shown in Figure 8a,b, respectively. In these figures, the reference and its main peak are shown using a solid blue line and green circle, respectively. In addition, the solid black curve represents input envelop. As shown in Figure 4, we use two parallel functions (Function (1) and Function (2)) to find the main peak based on the dynamic window; the main peak positions extracted using these functions are shown by red and blue circles on the envelope (black curve), respectively. Due to finding different positions for main peak 5 in the first instance envelope shown in Figure 8a, the comparison algorithm is applied and the peak position, which is highlighted in blue, is selected as the best position for the main peak. After that, the main peaks based on static window are also extracted, and the blue circle is considered a result of this process. Therefore, due to obtaining similar results in the processes of main peak extraction based on dynamic and static windows, the comparison algorithm is not reapplied for a second time. In another instance, regarding the envelope shown in Figure 8b, the results of two parallel functions based on the dynamic window are the same for main peak 6 that those shown by the blue circle in the envelope. Therefore, the comparison algorithm to compare the results extracted by two dynamic windows is not applied. While a different position, shown by red circle, is extracted from the static window, the comparison algorithm should be applied to select the best result between the dynamic and static windows. After comparison, the blue circle is selected as the best position. It should be noted that the threshold (input parameter) in the comparison algorithm is set to 6.

**Figure 9:**
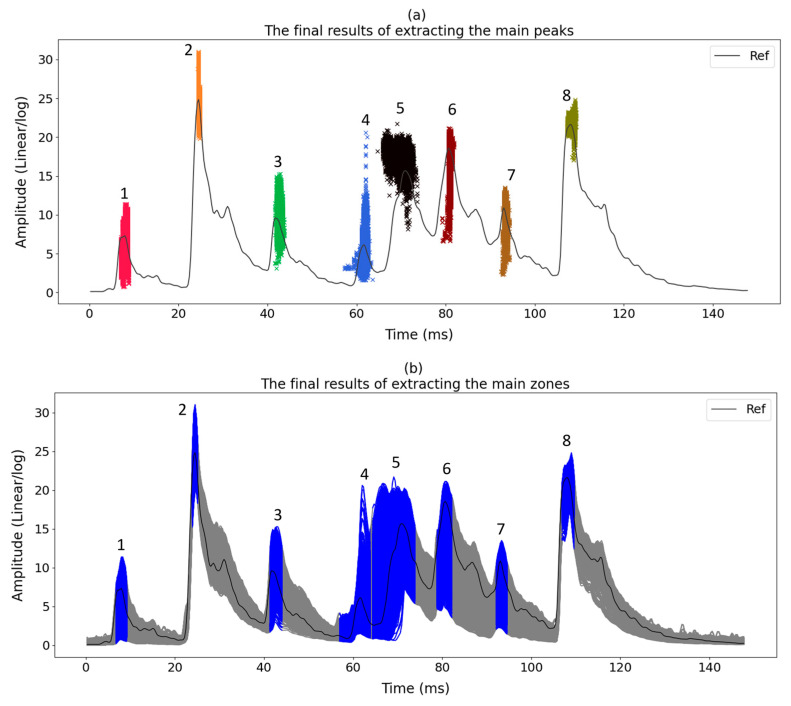
(**a**) Main peaks extracted in dataset T3A-odd. Note that the size and location of each zone highlighted in different colors are determined based on the location of the amplitude of the main peaks; (**b**) The related zones are highlighted in blue.

The results of the main peaks in each envelope are shown in Figure 9a. It is also possible to subdivide each dataset into several main zones. The size and location of each zone are determined based on the location of the amplitude of the main peaks shown in Figure 9a. It should be noted that the size of each zone can be computed using Equation (5). Figure 9b displays the main zones which are highlighted in blue. The location of each zone is also provided in Table 1.

### 5.2. Computing ED to Track Changes in Signal Envelopes over Time

In this section, ED is calculated between the reference signal and each envelope. This step is repeated twice. The ED is first calculated using the localization of main peaks. Secondly, EDs are computed using the samples for each zone as vectors in Equation (6). The average ED per year is computed to detect if there is a changing or evolving trend in the data.

**Figure 10:**
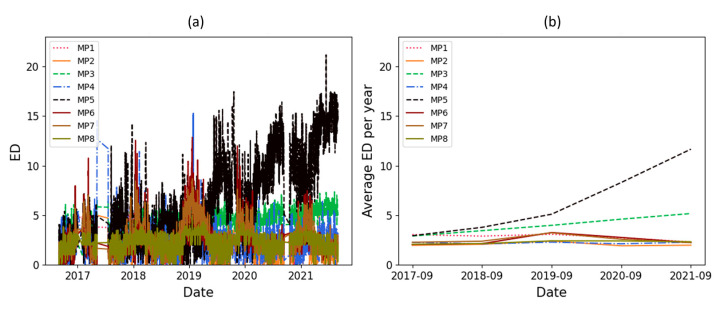
ED computed on the main peaks with respect to time (in dataset T3A-odd).

**Figure 11:**
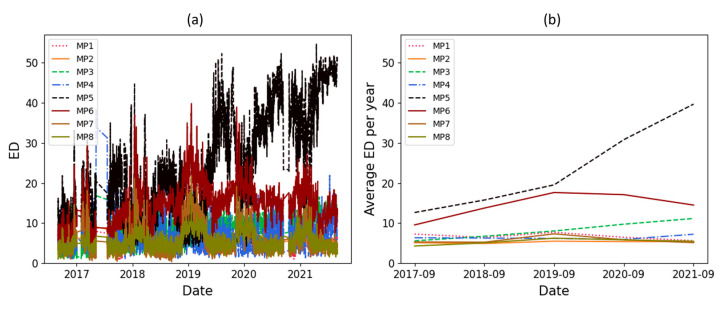
ED computed on the zone of the main peaks with respect to time (in dataset T3A-odd).

The ED results related to the amplitude of the main peaks and their zone are available in Figure 10 and Figure 11, respectively. In these figures, ED and the average ED per year are shown in individual subfigures. These figures show clearly the seasonal pattern and an increasing trend for one of the main peaks.

## 6. Discussion

**Figure 12:**
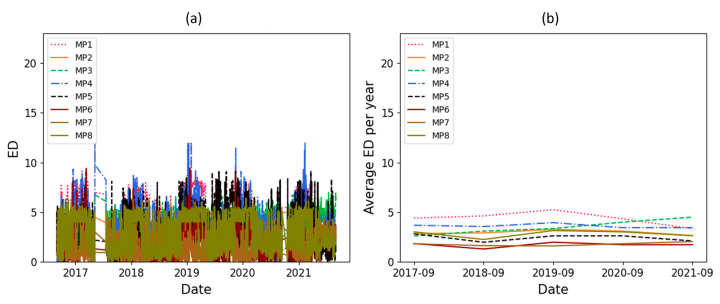
ED computed on the main peaks with respect to time (in dataset T3C-odd).

**Figure 13:**
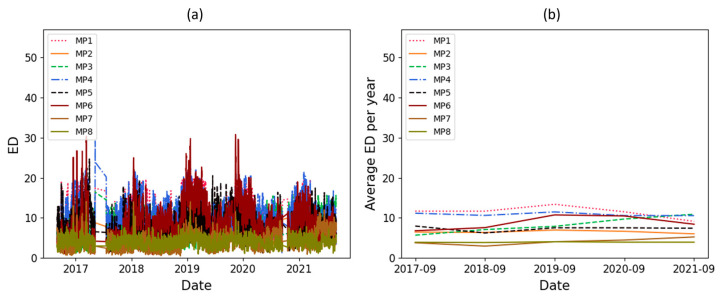
ED computed on the zone of the main peaks with respect to time (in dataset T3C-odd).

**Figure 14:**
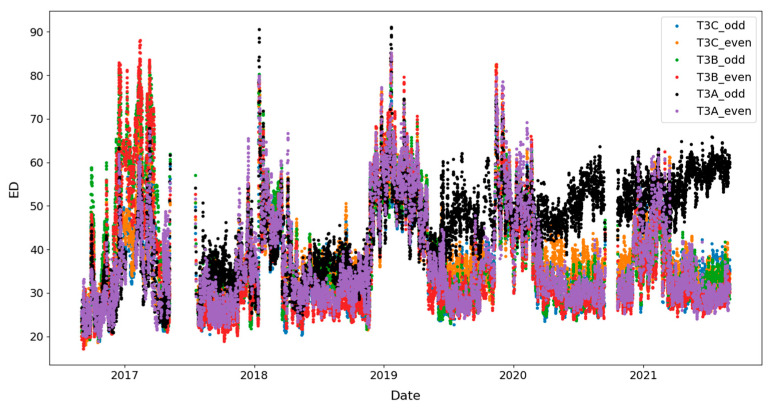
ED computed on the whole envelope for all studied datasets.

As mentioned, a significant increasing trend is seen in dataset T3A-odd. This trend is not visible in all the other datasets of the sister units located in the same substation, as shown in Figure 12 and Figure 13 for the T3C-odd dataset. Moreover, Figure 14 shows the ED values computed between the 500 samples of each envelope compared to the reference. As shown in this figure, dataset T3A-odd has a different pattern compared to the other datasets [23]. It should be noted that the already implemented algorithm in the experimental setup is based on the measured global ED (see Figure 14) and there is an alarm threshold based on the normal distribution of the recorded data. The developed algorithm in this paper, which is an effort to improve the current practice, utilizes other criteria to detect the fault, which is different than the already implemented algorithm. The proposed method aims to detect and locate the changes in the OLTC’s switching vibro-acoustic signal envelopes. This is a more in-depth analysis that supports the global trending made on the whole switching signature (Figure 14). According to this goal, the main peaks and zones containing the main peaks are extracted to detect the location of changes in vibro-acoustic signal envelopes. Figure 9a,b represent the locations of the main peak and zones in the T3A-odd dataset, respectively. The provided results indicate that the developed algorithms succeed in extracting features from vibro-acoustic signal envelopes despite the close proximity of the main peaks 4, 5, 6, and 7, and the observed displacement of main peak 5. After that, the ED metric is used to track changes in the main peaks and zones. The results of ED values related to the main peak and zones are presented in Figure 10 and Figure 11, respectively. As illustrated in these figures, part 5 (MP5 and Z5) has a pattern that differs from the other parts. It can be concluded that the source of the global changes in the T3A-odd dataset (as shown in Figure 14) is due to this specific part of the envelope.

A linear regression technique is used to determine if there is an increasing or decreasing trend in the ED’s computed values. This technique is applied to the average ED per year, and if the absolute coefficient of linear regression is greater than 0.5 and R^2^ is higher than 0.8, it indicates that there is a significant change over time. The coefficient value of linear regression related to the average ED per year for the main peaks and their zone are represented in Table 2. In this table, the main peaks and zones 3 and 5 are in bold because the thresholds (coefficient and R^2^) are exceeded, indicating that significant changes have been detected in these parts of the vibro-acoustic signal envelopes. Although the changes in main peak and zone 5 were clear upon a visual inspection of Figure 10 and Figure 11, the main peak and zone 3 also had a smooth change over time which was detected using a statistical test.

In future works, anomaly detection techniques supported by artificial intelligence will be explored to detect changes in the vibro-acoustic signal envelopes. In addition, researchers will aim towards a more in-depth understanding of the physical sources that create the main peaks, in order to achieve diagnostics after an anomaly is detected and improve the condition-based maintenance.

## 7. Conclusions

In this research work, a new methodology is developed to detect, locate, and track changes in envelopes of vibro-acoustic signals related to power transformers OLTCs. The developed method accomplishes this through two major steps: main peak extractions and ED computations. As such, the main peaks are extracted from the envelopes to detect and locate the changes in the envelopes. According to the locations of these main peaks, the main zones are also extracted from envelopes. Afterwards, the ED metric is utilized to compute the amount of similarity between the over-time monitored envelopes with the reference. In this regard, ED is computed on the main peaks location and on their corresponding zone. According to the ED values, significant changes were observed in one main peak and its zone. The linear regression technique was applied on the average ED per year to enable an automated detection. The results show that this methodology is not only able to detect changes in vibro-acoustic signal envelopes, but it also provides a more in-depth analysis of the location of these changes in the vibro-acoustic signal envelopes, and the rate of change over time. The developed algorithms were applied to six different real-life datasets obtained from experimental measurements and the main peaks and zones were extracted successfully. The provided results indicate that the developed algorithms succeed in extracting features from vibro-acoustic signal envelopes. Moreover, the success in feature extraction of different datasets indicates the developed algorithms’ reliability to be applied to every dataset of similar OLTC types.

## Figures and Tables

**Table 1:** Location of each main zone in dataset T3A-odd.

Zone Number	Location of Zone
1	From 6.5 ms to 9.2 ms
2	From 23.6 ms to 25.1 ms
3	From 41.1 ms to 44.0 ms
4	From 56.7 ms to 63.8 ms
5	From 64.1 ms to 73.9 ms
6	From 78.6 ms to 82.1 ms
7	From 91.9 ms to 94.5 ms
8	From 106.7 ms to 109.6 ms

**Table 2:** Linear regression coefficients related to the average ED per year computed for main peaks and zones in dataset T3A-odd. The absolute coefficient of linear regression that is greater than 0.5 and R^2^ is higher than 0.8 are in bold.

Main Peak and Zone Number	Coefficient (Main Peak)	Coefficient (Zone)
1	−0.16420971	−0.32365302
2	−0.01289195	0.10738021
**3**	**0.56964947**	**1.41485584**
4	0.00550331	0.14465934
**5**	**2.19588839**	**6.90356619**
6	0.1016055	1.3236921
7	0.01265612	0.02436068
8	0.07725418	0.31156756
